# Phytoestrogens **β**-Sitosterol and Genistein Have Limited Effects on Reproductive Endpoints in a Female Fish, *Betta splendens*

**DOI:** 10.1155/2014/681396

**Published:** 2014-02-23

**Authors:** A. C. Brown, L. M. Stevenson, H. M. Leonard, K. Nieves-Puigdoller, E. D. Clotfelter

**Affiliations:** ^1^Department of Biology, Amherst College, Amherst, MA 01002, USA; ^2^Graduate Program in Organismic and Evolutionary Biology, University of Massachusetts, Amherst, MA 01003, USA; ^3^Department of Embryology, Carnegie Institute of Washington, Baltimore, MD 21218, USA; ^4^Doctoral Program in Ecology, Evolution and Marine Biology, University of California, Santa Barbara, CA 93106, USA

## Abstract

Phytoestrogens are produced by plants and may cause endocrine disruption in vertebrates. The present study hypothesizes that phytoestrogen exposure of female Siamese fighting fish (*Betta splendens*) may disrupt endogenous steroid levels, change agonistic behavior expression, and potentially also disrupt oocyte development. However, only the pharmacologic dose of **β**-sitosterol had a significant effect on opercular flaring behavior, while we did not find significant effects of **β**-sitosterol or genistein on steroids or gonads. These findings are in direct contrast with previous studies on the effects of phytoestrogens in female fish. Results of the current study support previous work showing that the effects of phytoestrogen exposure may be less acute in mature female *B. splendens* than in other fish.

## 1. Introduction

Phytoestrogens are structurally similar to endogenous estrogens such as 17*β*-estradiol but are produced by plants. The most well-understood phytoestrogen action on animal physiology, due to ingestion or exposure to contaminated water, involves competitive binding to estrogen receptors. Because of this ability, some phytoestrogens have documented medicinal potential [1], but in uncontrolled conditions they may adversely affect reproduction [2–5]. Furthermore, phytoestrogens may also interfere directly with steroid biosynthesis, intracellular signaling, cell proliferation, and gene expression [6], which has raised concerns in the medical community about their safety [7].

Consequences of exposure to these compounds are still unclear, as phytoestrogens have been reported to have estrogenic as well as antiestrogenic effects on vertebrates [8]. Estrogen receptor signaling and its importance in influencing development, behavior, and reproduction is only partially understood [9], so the far-reaching impacts of phytoestrogen exposure, including the alternative modes of action, are difficult to predict. Generally, phytoestrogens are considered safe for humans at common exposure levels, such as those found in soy products [10], but the large-scale anthropogenic production of phytoestrogens in runoff from agricultural areas [11], wood pulp mill discharge [12], and sewage treatment plant effluent [13, 14] may still pose a threat to aquatic ecosystems.

Even subtle reproductive impairments due to phytoestrogens can have detrimental impacts on wild fish populations [15]. Reproductive problems caused by phytoestrogens include delayed oocyte maturation in female medaka (*Oryzias latipes*) at 750 and 30,000 ng genistein per fish [3, 16], increased egg mortality and larval deformation of brown trout (*Salmo trutta*) at 10 and 20 *μ*g L^−1^ mixed phytosterols found in pulp mill effluent [17], and confused sexual differentiation in mosquitofish (*Gambusia affinis holbrooki*) [18]. Furthermore, the effects of phytoestrogen exposure in fish are not limited to the direct effects of endocrine disruption; zebrafish (*Danio rerio*) embryos injected with genistein experience neural cell apoptosis in the hindbrain without binding to estrogen receptors [19]. Environmental concentrations of phytoestrogens have been reported to range from 1 to 41 *μ*g L^−1^ in some areas, with increasing concentrations correlated to the physiological effects mentioned above [20].

Abnormal behavior can be the first and most readily observable manifestation of endocrine disruption [21]. Phytoestrogens cause decreased sex and social behavior [4, 22, 23] and increased aggression and anxiety [22–24] in mammalian studies, while fish responses range from depressed agonistic behavior in Siamese fighting fish (*Betta splendens* Regan, 1910) [25] to male-typical courtship behaviors in female mosquitofish [18]. To fully characterize the effects of phytoestrogen exposure on this species, the current investigation considers the potential effects of short-term exposure to both environmentally relevant and pharmacological concentrations of genistein and *β*-sitosterol on mature female reproductive behavior and ovary development.

Previous research from our lab posited that Siamese fighting fish are an ideal system in which to investigate the effects of endocrine disrupting chemicals on behavior, as they exhibit well-characterized and easily quantifiable agonistic behaviors [25–27] and have been used previously in studies of *in vitro* toxicology [28, 29]. An early study from our lab supported male fighting fish as good candidates for demonstrating the ecological impact of phytoestrogens, as they showed a significant decrease in agonistic behaviors as a result of exposures [26]. However, in a subsequent physiological study, the only effect detected was decreased sperm count, with no observable decline in fertilization [30].

The present study hypothesizes that phytoestrogens will reduce endogenous sex steroid hormone levels, resulting in behavioral changes and delayed ovulation and oocyte development in female Siamese fighting fish. We use two common phytoestrogens, genistein and *β*-sitosterol, which can produce disruptive effects in animals via competitive binding of estrogen receptors [22, 31–33] and are often present in the same plants [34]. We predict that circulating hormone levels will decrease, resulting in reduced gonad mass and oocyte maturation delay. Behavior towards conspecific males may be suppressed. If the present study does not observe these changes, we may conclude that Siamese fighting fish are relatively tolerant of endocrine disrupting chemicals in their environment.

## 2. Materials and Methods

### 2.1. Ethics Statement

All animal protocols used in these experiments were approved by the Institutional Animal Care and Use Committee (IACUC) of Amherst College (Clotfelter).

### 2.2. Study Subjects and Phytoestrogen Exposure

Female *B. splendens* (*n* = 173) used in the following experiments were raised in captivity from domestic stock and obtained from a commercial supplier. Fish ranged in standard length from 24.33 to 38.85 mm and ranged in mass from 0.37 to 1.46 g. All fish were at least one year of age and sexually mature at the time of exposure. Each fish was individually housed in 800 mL of water in a 1 L glass beaker and fed to satiety with freeze-dried mosquito larvae once daily. Uneaten food was removed immediately with a bulbed syringe.

All the water used for housing and exposure tests was filtered using reverse osmosis and reconstituted to pH 6.5 and conductivity between 100 and 200 S cm^−1^. Water temperature was maintained at 19 degrees C in a climate controlled room. Dissolved oxygen was 10 ± 1.5 mg L^−1^. Phytoestrogens were dissolved in ethanol and resuspended in reconstituted reverse-osmosis water at the appropriate concentration for use in a semistatic exposure. Although phytoestrogens are relatively stable in the environment [35, 36], treatment and control water was changed and replenished with the appropriate concentration of phytoestrogens every day for 21 days to maintain exposure levels throughout the experiment [30]. The exposure duration of 21 days was selected because similar studies using other endocrine disrupting chemicals have shown significant negative effects on a range of fish species after 3-4 weeks of exposure [37–40].

Fish used to collect behavioral or hormonal data were placed in one of five treatment groups: negative control (ethanol vehicle only), 1 *μ*g L^−1^ genistein or *β*-sitosterol, and 1000 *μ*g L^−1^ genistein or *β*-sitosterol. For histological analyses, female fish (*n* = 73) were placed in the following groups: negative control (ethanol vehicle only), 100 *μ*g L^−1^ 17*β*-estradiol, 1 *μ*g L^−1^ genistein, 1 *μ*g L^−1^ *β*-sitosterol, and a mixture of 1 *μ*g L^−1^ genistein and 1 *μ*g L^−1^ *β*-sitosterol. The low doses (1 *μ*g L^−1^) are nominal concentrations within the range of those reported in nature [26]. The high doses (1000 *μ*g L^−1^) used in the behavioral and hormonal experiments are pharmacological concentrations. 17*β*-estradiol was used in the histology experiment because it is known to depress oocyte maturation and yolk deposition in female guppies (*Poecilia reticulata*) [41]. 17*β*-estradiol was omitted from the behavioral and hormonal work because it cross-reacts with the detection assay probe used here. Exposure levels were confirmed using high performance liquid chromatography as described previously [42].

### 2.3. Behavioral Studies

After the 21-day exposure period, 100 treated and control females were transferred to 2 L cubical aquaria containing treatment water and then visually presented with one of 14 live, untreated males in an adjacent aquarium. Previous studies have shown that transportation to a novel tank has no measurable effect on stress response in domesticated *B. splendens* [43]. The female fish were videotaped for 10 minutes and these recordings were scored for duration of three behavioral responses: latency to respond to the presence of the male, duration of opercular displays (tonic movement of the operculum and branchiostegal membrane), and duration of fin displays (erection of the dorsal and caudal fins). These displays are important parts of the agonistic and courtship repertoires of both female and male *B. splendens* [21, 25, 27]. Where *n* < 100, subjects were removed from the experiment because they died or showed signs of illness (*n* = 12 total, across all treatment groups).

### 2.4. Hormonal Studies

In order to measure how phytoestrogen exposure affected levels of endogenous hormones testosterone and 17*β*-estradiol excretion, we used a noninvasive technique commonly used in *B. splendens* and other fish to provide a proxy for plasma hormone levels [44]. After the 21-day exposure period outlined in the previous section, fish were placed in 400 mL of reconstituted reverse osmosis (RO) water (not treated with phytoestrogens) and visually presented with a male stimulus fish for 12 hours. Following this treatment, the subject fish were then isolated for 2 hours. Water (100–150 mL) was collected from each fish. Fish excrete hormones through their urine, feces, and gills, which can be extracted from the surrounding water. In brief, a sample of 50 mL water was drawn through SPE extraction columns, eluted in methanol, dried under nitrogen gas, and resuspended in enzyme immunoassay (EIA) buffer. Concentrations of the steroid hormones testosterone and 17*β*-estradiol in the water of exposed and control fish were measured using EIA assay kits (Cayman Chemical Co.). The antiserums used in these assays, while prone to interference under certain circumstances, are highly specific to the endogenous steroid they are designed to test and therefore do not detect excreted phytoestrogens. Steroid hormone amounts are reported as pg of detected hormone per mL sample water per gram of fish mass (pg/mL/g).

### 2.5. Histological Analyses

**Figure 1:**
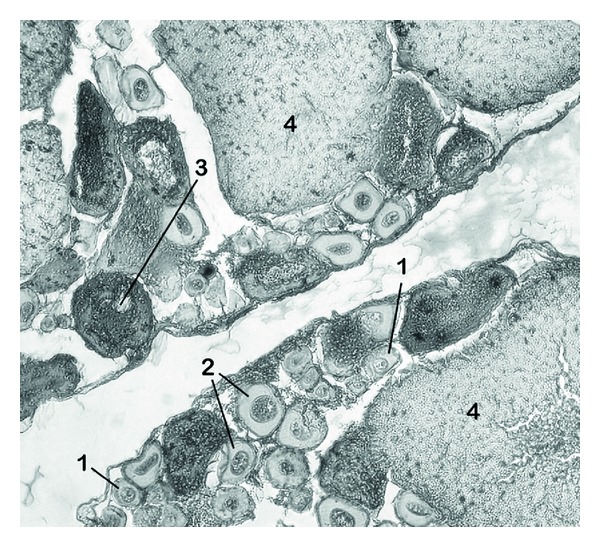
Female *B. splendens* gonad histological section under 400x magnification. Four distinct categories of oocyte maturation were identified: (1) one nucleolus present in the nucleus; (2) multiple nucleoli present and lipid droplets in the cytoplasm; (3) VTG globules in the periphery of the cytoplasm or layered in the zona radiata; (4) oocyte is large, full of VTG, with a nucleus at the oocyte periphery (or nucleus not visible due to slicing).

Semistatic exposures were conducted as described previously, and female fish were euthanized after the 21-day exposure period. Fish mass and ovary mass were used to calculate gonadosomatic index (GSI; measured as (gonad weight/(total body weight))∗100). After weighing, ovaries were fixed in Bouin's solution (Ricca Chemical Co.) for 24 hours. Following fixation, ovaries were dehydrated with ethanol, cleared with Hemo-D xylene substitute, and infiltrated with paraffin. The specimens were then embedded in paraffin and trimmed. Ovaries were cut into 6 *μ*m sections with a microtome and mounted on gelatin-coated slides. Sections were cleared with Hemo-D xylene substitute and stained with hematoxylin and eosin. Each 10th section was photographed under 400x magnification and scored using Image J software, version 1.43 [45]. Four stages of oocyte maturation were defined as described in Figure 1 [2] and counts of each cell type were performed. Final counts were converted to proportions in order to accommodate unequal numbers of histological slices.

### 2.6. Statistical Analyses

Data analysis for all experiments was performed using R (R Development Core Team, 2010). For variables that were not normally distributed, we used a log transformation to achieve normality. Steroid data were analyzed with ANOVA and Dunnett's post hoc tests. For analysis of behavioral data, we were unable to achieve normality of residuals through transformation, so treatment effects were estimated with robust regression using M-estimation from the MASS library [46]. Histological data met the assumptions of a multivariate analysis of variance (MANOVA), which we then used to analyze the data. Post hoc statistical power was calculated based on small, medium, and large effect sizes (*f*_2_) of 0.02, 0.15, and 0.35, respectively.

## 3. Results

**Figure 2:**
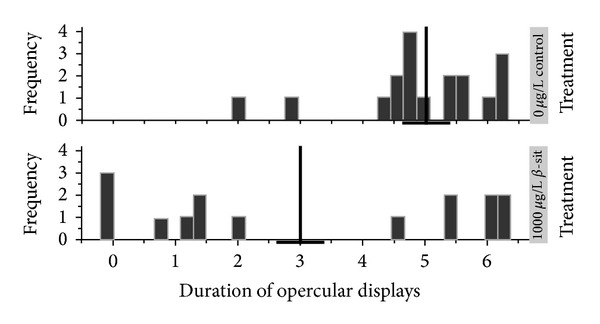
Female *B. splendens* exposed to 1000 *μ*g L^−1^ of *β*-sitosterol spent significantly less time engaged in opercular displays than fish in the control treatment group. Mean and standard error of the residuals of the log transformed data for each group are indicated by the vertical and horizontal crossbars, respectively. This figure also illustrates the presence of many extreme values that could not be resolved by transformation.

Raw behavioral data is presented in Table 1. Females treated with the pharmacologic dose (1000 *μ*g L^−1^) of *β*-sitosterol spent less time performing opercular displays than fish in the negative control group (*t*_(88)_ = −3.03, *P* = 0.002; Figure 2). Opercular flare duration was not affected in the environmentally relevant *β*-sitosterol treatment group (*t*_(88)_ = −0.86, *P* = 0.20) or either genistein treatments (1 *μ*g L^−1^: *t*_(88)_ = −1.61, *P* = 0.06; 100 *μ*g L^−1^: *t*_(88)_ = −0.34, *P* = 0.37). The latency of females to respond to conspecific males was not significantly affected by genistein (1 *μ*g L^−1^: *t*_(78)_ = 1.08, *P* = 0.85; 100 *μ*g L^−1^: *t*_(78)_ = 1.63, *P* = 0.95) or by *β*-sitosterol (1 *μ*g L^−1^: *t*_(78)_= 1.72, *P* = 0.95; 100 *μ*g L^−1^: *t*_(78)_= 0.79, *P* = 0.78). Similarly, duration of female dorsal and caudal fin displays was not significantly affected by genistein (1 *μ*g L^−1^: *t*_(84)_ = −0.39, *P* = 0.35; 100 *μ*g L^−1^: *t*_(84)_ = 0.66, *P* = 0.74) or *β*-sitosterol (1 *μ*g L^−1^: *t*_(84)_ = −0.31, *P* = 0.38; 100 *μ*g L^−1^: *t*_(84)_ = −0.68, *P* = 0.25).

Circulating levels of testosterone in female *B. splendens* were not significantly affected by exposure to genistein or *β*-sitosterol (*F*_4,85_ = 0.50, *P* = 0.73). Neither were levels of endogenous 17*β*-estradiol in female fish significantly affected by genistein or *β*-sitosterol (*F*_4,90_ = 0.41, *P* = 0.80). Our statistical power to detect small (0.02), medium (0.15), or large (0.35) effect sizes of phytoestrogen exposure on endogenous testosterone was 0.05, 0.16, and 0.71, respectively, while our power to detect effect sizes for estrogen were 0.05, 0.17, and 0.74.

For details on GSI and oocyte histology results, see Table 2. There was no effect of the phytoestrogen treatment or positive control on female GSI (*F*_4,73_ = 1.91, *P* = 0.12). Analysis of ovary development indicated that there was no effect of genistein, *β*-sitosterol, or the positive control 17*β*-estradiol on oocyte maturation (MANOVA; df = 60, maturation categories: (i) *P* = 0.23, (ii) *P* = 0.11, (iii) *P* = 0.18, and (iv) *P* = 0.71). Our statistical power to detect small (0.02), medium (0.15), or large (0.35) effect sizes of phytoestrogen exposure on ovary development was 0.05, 0.13, and 0.58, respectively.

## 4. Discussion

The current study predicted that waterborne phytoestrogens would decrease endogenous steroid levels in female *B. splendens*, because male *B. splendens *exposed to environmentally relavent concentrations of estrogens show decreased reproductive behavior and altered monoamine neurotransmitter activity in the brain [26, 42, 47]. But we found no effects of phytoestrogens on circulating levels of testosterone and 17*β*-estradiol in females. One effect of phytoestrogens on female behavior towards males was detected, but only at the pharmacologic dosage, and no effects were detected on gonadosomatic index (GSI) or oocyte development.

The biological significance of the present study's observation that a high dose of *β*-sitosterol depressed female opercular displays is difficult to interpret. While the pharmacological dose that produced the depression in flaring behavior was twice as concentrated as the highest levels of mixed phytoestrogens found in the environment [26], it is comparable to levels used in previous fish physiology studies [3, 16]. Opercular displays in male *B. splendens* are correlated with paternal care, and may be indicative of a male's physiological tolerance of hypoxia [48, 49], but the role of this behavioral display in determining the outcome of social interactions and fitness among female fighting fish is unknown. Nor did the present study detect an effect of phytoestrogens on endogenous hormones, even at the highest exposure levels. A few studies also suggest that different phytoestrogens may affect behavior through multiple pathways; for example, *β*-sitosterol may mimic cholesterol in addition to estrogen [50, 51]. These pose interesting questions for further research, but the present study cannot draw any conclusions about the mechanism that caused the depression in opercular display unaccompanied by hormonal disruption.

Histological examination revealed that ovaries contained a mix of oocytes at varying stages of maturation, but there was no difference in ovule maturation with respect to treatment group. Similarly, phytoestrogens did not cause a change in GSI. The nonsignificant results of the present study support the nonsignificant effects of genistein and *β*-sitosterol steroids and GSI data that we found previously in male *B. splendens* [30]; however, estrogenic compounds inhibit oocyte development [17, 52] and ovulation [2] as well as cause reductions in GSI [52] and gonad size [53] in other fish. It is possible that if we had included the pharmacologic doses (1000 *μ*g L^−1^) in the histological testing, we may have detected an effect of phytoestrogens on the gonads. However, as with the significant behavioral change, its biological significance would have been difficult to interpret.

The authors do not interpret this finding as an indication that anthropogenic phytoestrogen concentration in waterways is safe for fish. In addition to fish as important indicators of ecosystem health [54], the health of waterways and wild populations of fish are inextricably linked to human health through the consumption of fish [55] and fish products, such as fish oil capsules [56]. The results of the present study are consistent with previous findings that endocrine disrupting compounds may have a reduced impact on steroid levels when exposure occurs in adulthood or over a short duration [57]. Greater exposure duration than 21 days or exposure to combinations of other chemicals in wood pulp mill effluent might produce an effect, as suggested by other researchers [58]. Although previous work found decreased sperm quality [42] and aggression [26] in male* B. splendens* exposed to phytoestrogens, the results of the present study reflect the assertion made previously [30] that *B. splendens* may be more resistant to phytoestrogens than previously thought.

## Figures and Tables

**Table 1:** Behavioral results from fish exposed to phytoestrogens or control. Means and standard errors are expressed in seconds.

	Vehicle only	1 *μ*g/L *β*-sitosterol	1 *μ*g/L genistein	1000 *μ*g/L *β*-sitosterol	1000 *μ*g/L genistein
Fin display	552.8 ± 74.1	495.0 ± 189.9	530.4 ± 124.6	513.1 ± 127.6	563.0 ± 79.6
Opercular flare	215.4 ± 169.3	155.5 ± 163.8	125.6 ± 139.7	159.5 ± 201.7	195.3 ± 151.0
Response latency	10.9 ± 15.2	50.6 ± 64.8	69.6 ± 117.9	13.69 ± 8.77	55.2 ± 89.0

**Table 2:** Histology and GSI measurements of adult female *B. splendens* across steroid and phytoestrogen exposures.

	Vehicle only	E2 (100 *μ*g/L)	Genistein (1 *μ*g/L)	*β*-Sitosterol (1 *μ*g/L)	Gen (1 *μ*g/L) *β*-sit (1 *μ*g/L)
GSI *F*_73_ = 1.90, *P* = 0.12	0.08 ± 0.03	0.07 ± 0.03	0.06 ± 0.03	0.08 ± 0.03	0.10 ± 0.034
Oocyte type (i) *F*_60_ = 1.48, *P* = 0.22	0.31 ± 0.13	0.28 ± 0.14	0.29 ± 0.14	0.25 ± 0.11	0.37 ± 0.12
Oocyte type (ii) *F*_60_ = 1.73, *P* = 0.16	0.37 ± 0.14	0.38 ± 0.11	0.41 ± 0.14	0.42 ± 0.15	0.37 ± 0.14
Oocyte type (iii) *F*_60_ = 1.17, *P* = 0.17	0.15 ± 0.05	0.19 ± 0.04	0.17 ± 0.04	0.17 ± 0.03	0.16 ± 0.03
Oocyte type (iv) *F*_60_ = 0.45, *P* = 0.77	0.16 ± 0.11	0.15 ± 0.08	0.13 ± 0.09	0.16 ± 0.03	0.17 ± 0.07
